# Maize Tassel Detection From UAV Imagery Using Deep Learning

**DOI:** 10.3389/frobt.2021.600410

**Published:** 2021-06-09

**Authors:** Aziza Alzadjali, Mohammed H. Alali, Arun Narenthiran Veeranampalayam Sivakumar, Jitender S. Deogun, Stephen Scott, James C. Schnable, Yeyin Shi

**Affiliations:** ^1^ Department of Computer Science, University of Nebraska-Lincoln, Lincoln, NE, United States; ^2^ Department of Computing, Community College, Prince Sattam Bin Abdulaziz University, Al Kharj, Saudi Arabia; ^3^ Department of Biological Systems Engineering, University of Nebraska-Lincoln, Lincoln, NE, United States; ^4^ Department of Agronomy & Horticulture, University of Nebraska-Lincoln, Lincoln, NE, United States

**Keywords:** phenotyping, object detection, flowering, faster R-CNN, CNN

## Abstract

The timing of flowering plays a critical role in determining the productivity of agricultural crops. If the crops flower too early, the crop would mature before the end of the growing season, losing the opportunity to capture and use large amounts of light energy. If the crops flower too late, the crop may be killed by the change of seasons before it is ready to harvest. Maize flowering is one of the most important periods where even small amounts of stress can significantly alter yield. In this work, we developed and compared two methods for automatic tassel detection based on the imagery collected from an unmanned aerial vehicle, using deep learning models. The first approach was a customized framework for tassel detection based on convolutional neural network (TD-CNN). The other method was a state-of-the-art object detection technique of the faster region-based CNN (Faster R-CNN), serving as baseline detection accuracy. The evaluation criteria for tassel detection were customized to correctly reflect the needs of tassel detection in an agricultural setting. Although detecting thin tassels in the aerial imagery is challenging, our results showed promising accuracy: the TD-CNN had an F1 score of 95.9% and the Faster R-CNN had 97.9% F1 score. More CNN-based model structures can be investigated in the future for improved accuracy, speed, and generalizability on aerial-based tassel detection.

## 1 Introduction

It is estimated that the world population will exceed 9 billion by 2050 and that current agricultural yields will need to grow as much as 50% per unit of land in order to insure food security by that year. Achieving this goal requires the development of high yielding, more stress tolerant, and more resource use efficient crop varieties. In modern breeding programs, data on genetic markers are combined with measurements of traits under field conditions to selected improved lines. Advances in sequencing data have substantially lowered the cost and increased the throughput of obtaining genetic marker information for candidate crop lines. However, phenotyping remains slower and more costly, and hence is often the rate-limiting step with hundreds to thousands of new lines waiting to be phenotyped and selected through multiple stages of breeding programs.

Flowering is a critical landmark in plant development, where the plant’s energy turns, in whole or in part, from growing more leaves in order to capture more energy to producing seeds and/or fruits. Maize is a monecious plant species with separate and specialized male and female inflorescences—flower-bearing structures—the tassel and ear, respectively. Tassels are produced at the top or above the canopy and pollen from the male flowers. These tassels must land on the silks produced by the female flowers of the ear for the grain to develop. Even moderate stress from heat or lack of water during the short flowering window can substantially reduce pollen production and yield potential in maize. The timing of male and female flowering is also under the control of at least partially distinct genetic architectures. In the wild, asynchronous male and female flowering can increase the likelihood of outcrossing. However, in agricultural production where an entire field is likely to be planted with a single variety in a single day, asynchronous flowering dramatically reduces yield potential by reducing the number of female flowers which receive pollen from male flowers.

Flowering time is a selection target in developing new maize varieties for two reasons. The first reason is to ensure that flowering timing is a match for the target environment. Early flowering would cause a large amount of photosynthetically active light energy waste, while too late flowering might kill the crop, if the season is changed before it can mature. The second reason is to ensure that a new candidate variety has good synchronization of male and female flowering in order to produce the maximum amount of grain. Currently, maize breeders manually score the flowering timing for hundreds or thousands of candidate lines by walking the field each day during the flowering season, which is extremely time and labor consuming (Smith et al., 2005). If we can rapidly identify the plots with tassels on the top part of the canopy, checking for silking down in the canopy can only be made over those targeted plots with a dramatic reduction of labor and time. This rapid identification of tassels on the top part of canopies would save breeders time and effort by allowing them to only check those targeted plots.

Maize tassels are thin-branched structures with individual branches several millimeters wide. While in some varieties the tassels produce anthocyanin and become purple as a result, in most commercial maize the tassel will be yellow to green in color, creating poor contrast with the leaf canopy below it. Most previous works on maize tasseling detection were using ground platforms (Zou et al. 2020); Shete et al. 2018). They capture images in ultra-high resolution but take a long time to cover the whole field, and have the difficulties of handling plants at different heights (Lu et al., 2017). The advent of low-cost and low-altitude unmanned aerial vehicles (UAVs) enable automated applications in agriculture to facilitate smart farming techniques. It is now possible to obtain aerial imagery with sub-centimeter spatial resolution for detecting small objects such as maize tassels (Sankaran et al., 2015; Shi et al., 2016). Advances in computer vision, particularly deep learning models for object detection, are promising for obtaining accurate image classification and localization of UAV images (Penatti et al., 2015) in agricultural biotechnology.

In this study, an automated approach to detect maize tasseling from UAV imagery was developed using deep learning techniques for 500 genotypes (750 plots) in a 2-ha breeding field. We compared a customized framework for tassel detection based on convolutional neural network with an off-the-shelf Faster R-CNN object detection model. Although our dataset were collected from fields in Nebraska, the methods developed in this study with low cost and high accuracy have great potentials to be easily adapted and integrated for field-based high-throughput maize tasseling detection.

## 2 Related Work

In this section, we reviewed various state-of-the-art machine/deep learning and object detection methods for plant-sensing applications.

### 2.1 Classical Machine Learning Applications in Plant Sensing

Maize tassels detection has been recently studied with other machine learning algorithms. For example, Kurtulmuş and Kavdir, (2014) employed ground-based imagery to detect the locations of the tassels using the support vector machine (SVM) classifier. They manually captured a small dataset of 46 high-resolution RGB images of maize canopy and classified pixels into tassel or non-tassel by extracting color information using SVM with a maximum accuracy of 81.6%. Lu et al. (2015) developed segmentation and an SVM-based approach to detect maize tassels by converting the RGB images (taken by a camera fixed on a pole) to saliency color space, and the potential regions with tassels were identified with an accuracy of 90.38%. Another study by Mokhtar et al. (2015) used SVM with a linear kernel to identify the powdery mildew disease of tomato leaves using images of thermal and stereo visible light.

Tang et al. (2011) applied image processing to identify maize tassel using image color space, and segmentation was to extract the part of maize tassel. After image preprocessing, they used segmentation algorithm based on HSI color space and region growing to extract and recognize the maize tassel. Reyes et al. (2017) developed a method based on image-processing techniques to estimate the leaf nitrogen concentration. They assigned SPAD unit values with its representative color value to every leaf. They then used k-means algorithm to segment the leaf images, generating a variable number of classes, for leaves or background, depending on the particular leaves’ color features. Another study (Xie et al., 2017) used k-nearest neighbor (KNN) and C5.0 to classify different types of samples to identify healthy and gray mold–diseased tomato leaves. Huang (2007) used back propagation neural network as their machine learning algorithm on RGB images to identify the bacterial soft rot, Phythopthora black rot, and bacterial brown spot diseases of Orchid (Phalaenopsis).

### 2.2 Deep Learning Applications in Plant Sensing

In classical machine learning methods, manual feature engineering process is required, while this process is done automatically in deep learning models. Deep learning has the advantage of using pretrained image classification models for speeding the feature extraction process for object detection, and still manages to achieve higher performance efficiently. Deep learning models outperform the classical machine learning methods for large datasets, which is the case of this study where high-resolution images were used. Kamilaris et al. provided a comprehensive survey of deep learning in agriculture (Kamilaris and Prenafeta-Boldu, 2018), which showed that there are many literature works done in applying deep learning in the agriculture domain for plants types classifications, disease detection, fruits counting, and others. They showed a strong promise of deep learning approaches on prediction applications in agriculture. Mohanty et al. (2016) trained a convolutional neural network on ground-based publically available images of diseased and healthy plant leaves to predict their species and disease type, if unhealthy.

Another related literature is TasselNet (Lu et al., 2017), where they used the same dataset as Sa et al. (2018) to classify the tassels using a CNN architecture, and then count the tassels using a regression algorithm. The images were taken from the side view from a ground-based platform. Similarly, Rahnemoonfar and Sheppard. (2017) counted the number of tomatoes using the CNN architecture and the regression algorithm. Their training was performed on images of the synthetic tomato plants, and then the testing was done on images of real tomato plants. In the Sa et al. (2018) study, micro aerial vehicle was used for semantic weed classification and detection. For this, they used a pixel-wise dense CNN to segment the image and trained the classifier. Deep learning approaches have also started to be deployed on aerial imagery for different applications (Zhu et al., 2017) though plant classification and geographical localization using these tools is still an emerging area. Penatti et al. (2015) applied CNN on aerial images to classify coffee in the Brazilian Coffee Scenes dataset. Similar studies on agricultural aerial images classification that employ deep learning techniques, CNN specifically, are Hung et al. (2014); Kussul et al. (2017); Milioto et al. (2017) and Sa et al. (2018).

While all the previous studies used ground-based platforms to collect their images, this study focused on aerial data and deep learning models to automate the agricultural procedures for the farmers. Our work builds on some of the ideas for classifying the maize tassels presented there and enhancing those approaches by identifying the tassels location, hence tassels detection. The object detection plays an important role in plant sensing to detect disease, pests, weeds, and help identify flowering stage for different crop types. And with the growing availability of numerous amount of agricultural data, the optimal approach to detect any object using deep learning models as they converge perfectly with big data.

### 2.3 Object Detection Models and Their Applications in Plant Sensing

Sliding window to generate region proposals and then classifying the objects was one of the leading classic methods for object detection in computer vision. One of the first efficient object detection methods was developed in 2001 by Viola and Jones (2001), where they use AdaBoost (Freund and Schapire, 1999) to select features of face images with the Haar basic function, and finally combine the classifiers into a cascade. Dalal and Triggs. (2005) invented histograms of oriented gradients (HOG) for pedestrian detection. They use HOG to describe the features. Those hand-crafted feature extraction methods fail to generalize for images with more distracted backgrounds.

#### 2.3.1 Object Detection Using Deep Learning

Object detection using deep learning consists of two stages, classification and localization. The classification was done using the convolutional neural networks (CNNs) to predict the objects in an image. CNNs are the basic building blocks for most of the computer vision tasks in the deep learning era. This CNN algorithm learns the patterns like vertical edges, horizontal edges, and round shapes, to recognize the object in the image. The convolution refers to the mathematical combination of two matrices to produce a third function merging two sets of information. In a CNN, the convolution is performed on the input data with the use of a filter to then produce a feature map (LeCun et al., 2010). The input images and their subsequent outputs are passed from a number of such filters, thus called deep learning. The second stage for the object detection is localization, where we not only want to know whether the object exists in the image but also where exactly the object is. Object localization algorithms label the class of an object and draw a bounding box around the position of the object in the image.

Deep learning for object detection has been advancing quite fast, one of the earlier advances was called OverFeat (Sermanet et al., 2014). They trained the CNN with a multiscale sliding window algorithm and then predict the box coordinates for the localization task for each object. R-CNN (Girshick et al., 2016) was then proposed by Girshicket al. after OverFeat. Their approach extracts possible objects using selective search as a region proposal method, then extracts features from each region using a CNN, and finally classify each region with SVMs. Then the same group introduced the Fast R-CNN, which uses the same region proposal of R-CNN and then apply the CNN on the complete image and use region of interest (RoI) pooling on the feature map with a final fully connected network for classification and regression. Another object detection method called “You Only Look Once” was proposed after Fast R-CNN by Redmon et al. (2016); it uses the fully connected layers after the feature extractor to predict the coordinates of bounding boxes directly. Subsequently, the third iteration of R-CNN, the Faster R-CNN was used as published by Ren et al. (2015). It replaced the selective search region proposal method with the region proposal network (RPN) which itself consists of a classifier and a regressor. The classifier uses anchors to slide a window over the feature maps and classify the objects based on the ground truth, so it determines the probability of a proposal having the target object for the regression to regress the coordinates of the proposals. After Faster R-CNN, two main object detection models were introduced. Single shot detector (SSD) (Liu et al., 2016) and the region-based fully convolutional networks (R-FCNs) (Dai et al., 2016).

We selected Faster R-CNN as one of the two models for tassel detection in this study due to its well-recognized high performance on high-resolution images and its ease of implementation. We considered its performance as baseline accuracy for our study.

#### 2.3.2 Object Detection Application in Plant Sensing

Object detection models (classification and localization) have been used by Bargoti and Underwood. (2017) and Sa et al. (2016), where Faster R-CNN network was employed to detect fruits from ground images. Another study by Fuentes et al. (2017) applied three different deep learning object detection algorithms including Faster R-CNN to ground images of tomato plants to recognize different disease and pets on those plants. They concluded that Faster R-CNN performed better in general. Pound et al. (2017) studied the application of CNN on plant phenotyping using images collected by a ground level camera, and they show promising results on classification and localization. They reported 97% accuracy on their model and thus emphasize on the feasibility of deep learning to solve challenging agricultural problems.

Object detection with deep learning methods in remote sensing images is the area directly related to our work. Some of others relevant work in this area include the following studies by Radovic et al. (2017), Božić-Štulić et al. (2018), and Milioto et al. (2017). Remote sensing data and deep learning methods have been put to other usage, for example, estimating the geolocation of ground images by extracting features from UAV images (Zhai et al., 2017) or detecting vehicles from aerial imagery (Sommer et al., 2017). Other applications are aerial images and CNN for cotton bloom detection by first training the CNN to learn to predict the blooms in images, then a motion method was used to obtain their locations from the aerial images, which were used to count the blooms and to monitor the flowering growth (Xu et al., 2018).

More object detection applications on plant sensing examples, specifically sorghum head detection, are the work of Ghosal et al. (2019); Guo et al. (2018). Ghosal et al. (2019)proposed a weakly supervised deep learning framework for sorghum head detection and counting from UAV imagery. They trained a CNN model to perform synthetic annotation. While Guo et al. (2018) proposed an image processing method to detect and count the number of sorghum heads from UAV imagery, and they verified their performance using a segmentation method. While similar to tassel detection, sorghum head is essentially a maize tassel covered in seeds, but the sorghum head is still an easier problem because they are thicker and have a lot more color contrast with the leaf canopy.

On rice panicle detection, Hayat et al. (2020) applied the Bayesian learning method to perform rice panicle segmentation with UAV optical images. They used an unsupervised learning approach to detect the required features to replace the training phase, using the Markov chain Monte Carlo (MCMC) method with Gibbs sampling. Moreover, Chandra et al. (2020) proposed a semiautomated annotation method for cereal crops panicle detection. Their method is useful to reduce labeling costs and showed positive results on two publicly available cereal crop datasets—Sorghum and Wheat, saving around 50% of labeling time.

As for deep learning–based models for maize tassel detection, very few studies were conducted so far using high-resolution aerial imagery for tassel detection. Some studies were done using other types of imagery data that were either available online (Penatti et al., 2015) or collected indoor or from ground-based platforms. We found only one study so far which applied Faster R-CNN to detect maize tassels from UAV imagery conducted by Liu et al. (2020). A keras-based Faster R-CNN model was applied to detect maize tassels from UAV images using images with 600 × 600 pixels to train the model, and results of different networks were compared for feature extraction and for different sizes of the anchor for object detection. Very promising prediction accuracy ranging from 87.94–94.99% was obtained when experimenting with different network parameters. Our work adopted a similar approach, in which we aimed to use high-throughput aerial imagery to help breeders to locate the tassels in field. Yet, we used images taken at a relatively higher altitude by a lower resolution and lower quality RGB camera that came with the UAV. Additionally, the tassels of the particular maize varieties in our trials were not as distinct as the background canopy, especially the leaf veins, which largely increased the challenging level.

### 2.4 Paper Contribution

This study applied object detection deep learning models for maize tassel detection from UAV images. We developed and compared two methods for automatic tassel detection based on imagery collected from a UAV using deep learning models. The first approach is a customized framework for tassel detection based on convolutional neural network. The other method is a state-of-the-art object detection technique of the faster region–based CNN (Faster R-CNN) model to detect tassels using bounding boxes along with coordinates to identify the location of the tassel on the map for the breeders.

This study filled the gap of few studies so far using deep learning–based models for maize tassel detection using aerial imagery taken from UAV-based low-cost low-quality RGB cameras. The main aim of this work was to provide a practical way to facilitate large-scale maize breeding by detecting the maize tassels automatically instead of the manual scoring process. Another contribution of this work was the customized evaluation metrics for the models that serves more appropriately for object detection in the agricultural setting in which the ground truth is difficult and impossible to be labeled exhaustively and accurately. This work can eventually contribute to increase the breeding efficiency to help solving the world hunger.

## 3 Dataset

### 3.1 UAV Image Collection

**FIGURE 1:**
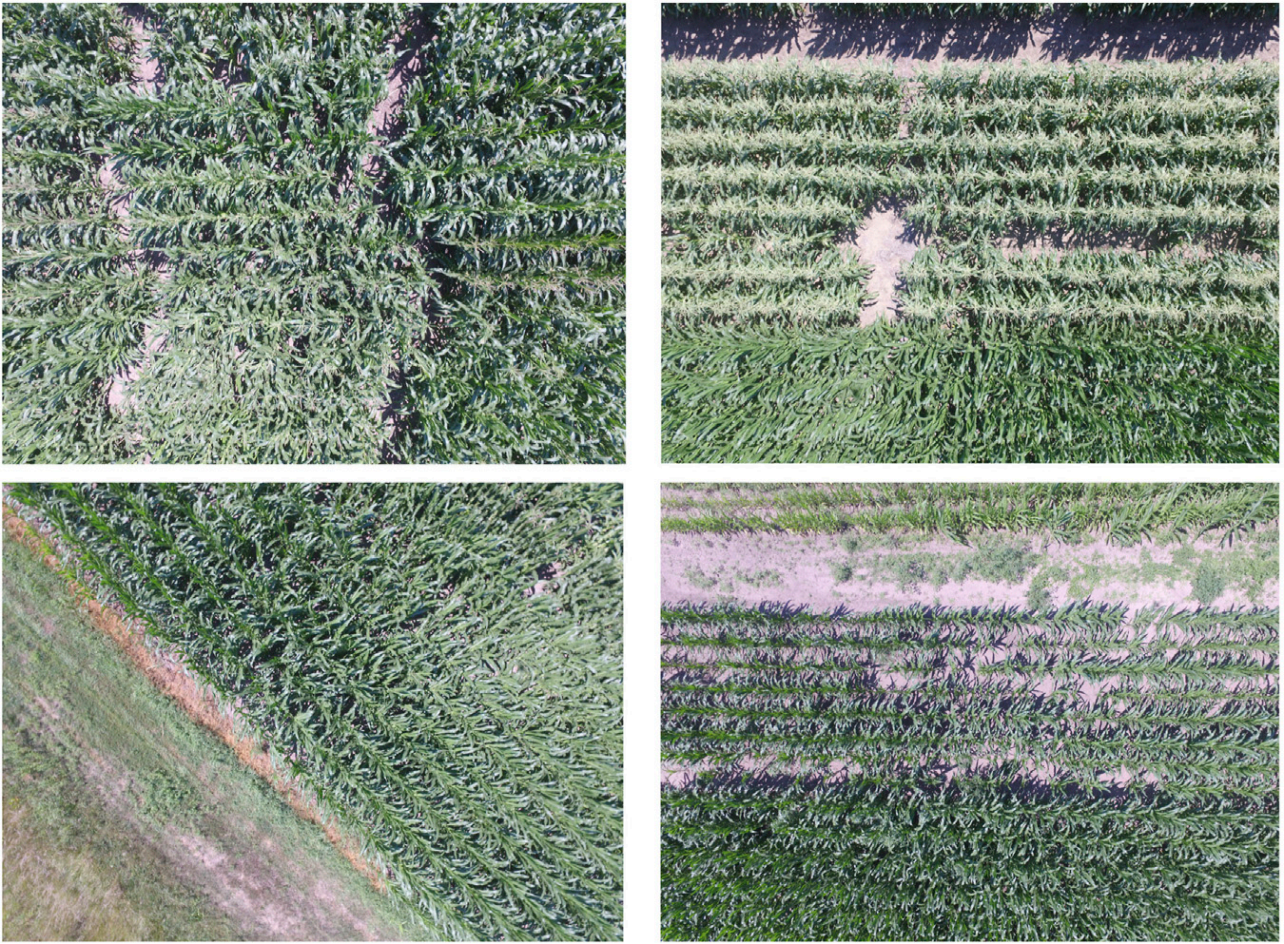
[Best viewed in color] Examples of the aerial images of the maize fields dataset employed in this study.

Image data employed in this study was collected from a maize variety field trial conducted at the University of Nebraska’s Eastern Research and Extension Center, near Mead, Nebraska. Images were collected using a DJI Phantom 3 Professional UAV with a 12.4 megapixel camera and 1/2.3″ CMOS sensor. The UAV was flown at an altitude of 20 m above ground level with around 90% forward overlap and 85% side overlap. Images were collected in July of 2017, fifty-four days after planting when a significant proportion of the research varieties had begun to flower. Figure 1 illustrates examples of the aerial image dataset including maize tassels, employed in this study.

### 3.2 Image Preprocessing

Raw images were of high resolution (3000 × 4000 pixels), which presented challenges both for accurate labeling and the computational resources needed to process and identify tassels. Each raw image was divided into subimages of 1000 × 1000 pixels without overlap resulting in a 3 × 4 grid of smaller images from each raw image, which were then fed to labeling process and the deep learning models.

**FIGURE 2:**
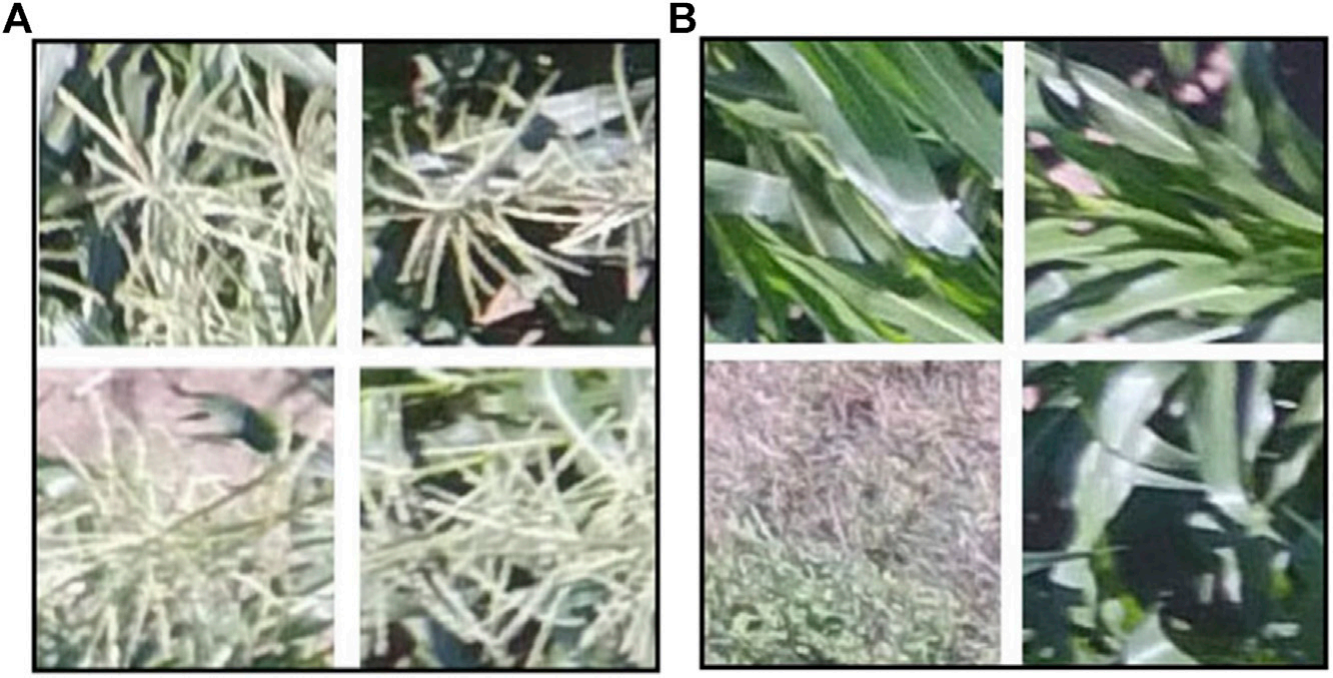
[Best viewed in color] Examples of the dataset used for the TD-CNN model: (a) “tassel” class images (b) “no_tassel” class images.

The locations of tassels were manually annotated in a set of 2000 of these smaller 1000 × 1000 subimages by using Labellmg (Lin, 2015), a labeling tool, to draw bounding boxes around each tassel and storing this information in an xml file associated with each image. For the Faster R-CNN model, 80% of the annotated subimages were used as training data and the remaining 20% were used for testing. For the positive class of TD-CNN model, smaller patches of 128 × 128 pixels were further extracted from the subimages that were previously annotated as “tassel” to form the new tassel class (Figure 2A). This was done automatically using a Python script. This size of the patches were chosen so that it is large enough to include a tassel with minimal background. Patches of “no_tassel” for the TD-CNN model were directly cropped from the UAV raw images (3000 × 4000 pixels), which had no tassels at all and had a variety of background such as grass, soil, and leaves but no tassels (Figure 2B). This was also done automatically using a Python script. Following the same approach employed for the Faster R-CNN model, 80% of the annotated patches were used as training data and the remaining 20% were used as testing data for the TD-CNN model.

## 4 Object Detection Models

In this study, we developed two object detection models to detect tassels from UAV imagery. One was a customized CNN-based tassels detection model, and the other was an off-the-shelf Faster R-CNN model. Any object detection model is basically composed of classification and localization. For the classification process, both TD-CNN and Faster R-CNN models used transfer learning. Transfer learning is a deep learning methodology that helps in transferring knowledge learned from classifying previous dataset to a new one (Pan and Yang, 2010). For most deep learning models, the availability of large dataset is essential in producing an efficient classification model that learns all the different features of the dataset. Since this big amount of data are not always available, transfer learning enables the use of a model which was already trained on a big dataset and thus have a strong general feature extraction base. Therefore, just the last layer of the deep model will be trained to adapt the new dataset features. Some examples of those pretrained deep learning architectures that can be used for transfer learning are Inception v3 (Szegedy et al., 2016) and ResNet50 (He et al., 2016) models. More details of each detection model is given in the following sections.

### 4.1 Tassel Detection CNN

**FIGURE 3:**
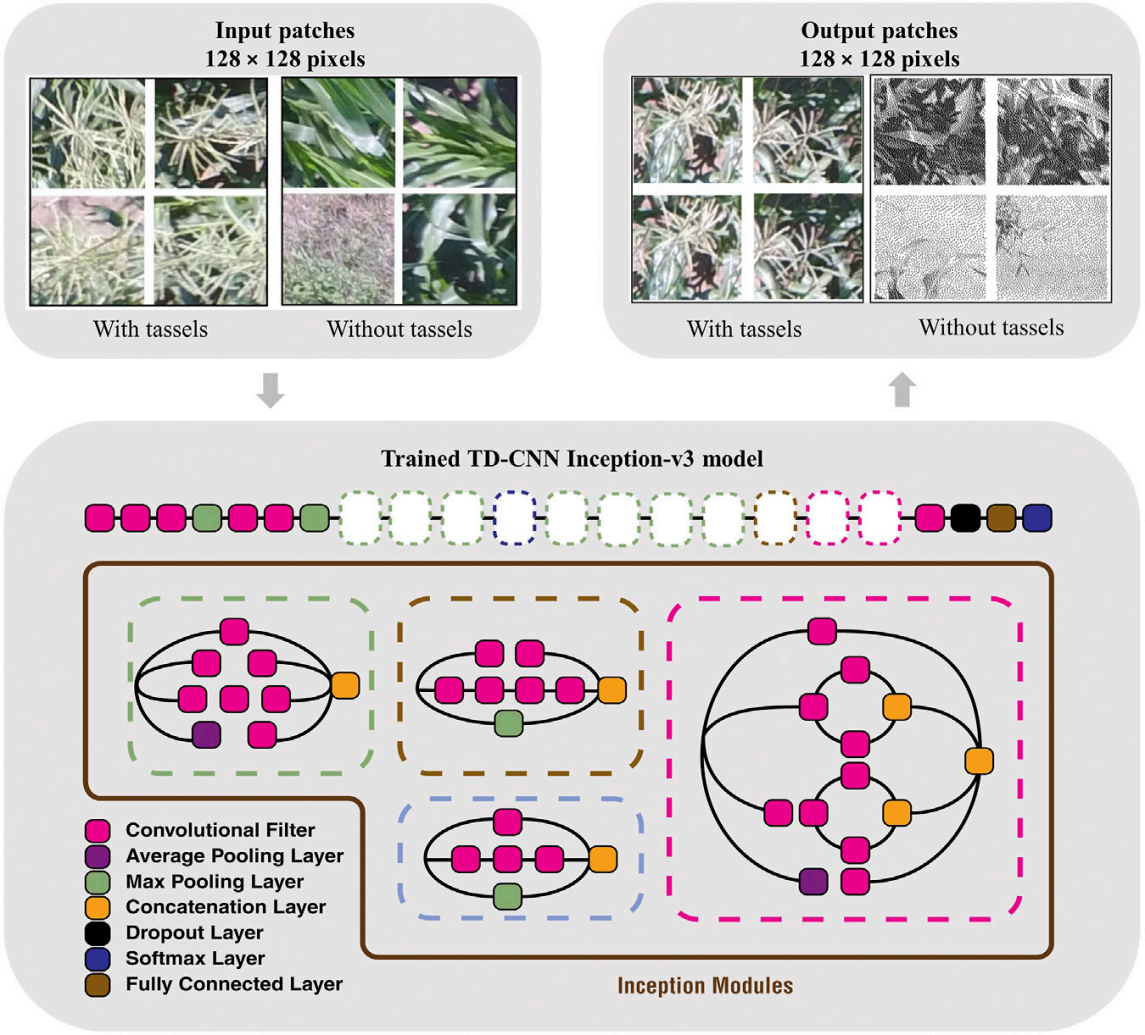
[Best viewed in color] Tassel detection with TD-CNN trained with the Inception v3 model.

We developed tassel detection CNN, a novel tassel detection model to detect tassels objects with complex shape in high-resolution remote sensing images as shown in Figure 3. The images were first divided into smaller patches of 128 × 128 pixels and labeled as two classes: tassel and not_tassel. Then a CNN model was used to classify the images. Finally, the classified output was mapped to the larger original images. The trained CNN model used for tassel classification was based on Inception v3 (Szegedy et al., 2016), a pretrained deep learning model. Inception v3 is able to learn the most important features in different kinds of images since it is pretrained on a large-scale hierarchical dataset called ImageNet. The default input size for Inception v3 was adapted from 299 × 299 pixels to 128 × 128 to match the size of our input patches. We believe that a patch of size 128 × 128 is better in capturing single or few tassels, rather than a larger patch size of 299 × 299. The last layer of the Inception v3 was retrained using tfClassifier (Pai, 2018) to fine-tune its classification on our tassels dataset. For the localization process, as shown in Figure 3, the TD-CNN model keeps the patches, which were classified as tassel, in their original input color. Alternatively, the model converts the patches that were classified as not_tassel to grayscale. We then mapped this output back to the larger image to detect and locate the regions containing the tassels. Since the breeders have the GPS information of the breeding plots, they can physically locate the tassels from the UAV images metadata and decide if flowering has started in a breeding plot.

### 4.2 Faster R-CNN for Tassel Detection

Faster R-CNN (Ren et al., 2015) was developed to detect objects in images using region proposal network (RPN). RPN is mainly a convolutional neural network that classifies and localizes objects of interest. Faster R-CNN is adaptable for accepting large-size images, making it a good candidate for large UAV images. In this study, the Faster R-CNN object detection model was used as a method to detect the maize tassels from UAV images. The Faster R-CNN is an efficient R-CNN (Girshick et al., 2016) since it internally processes the region proposal network algorithm, which uses anchors to scale the proposed region of interest that has a high probability to contain an object.

**FIGURE 4:**
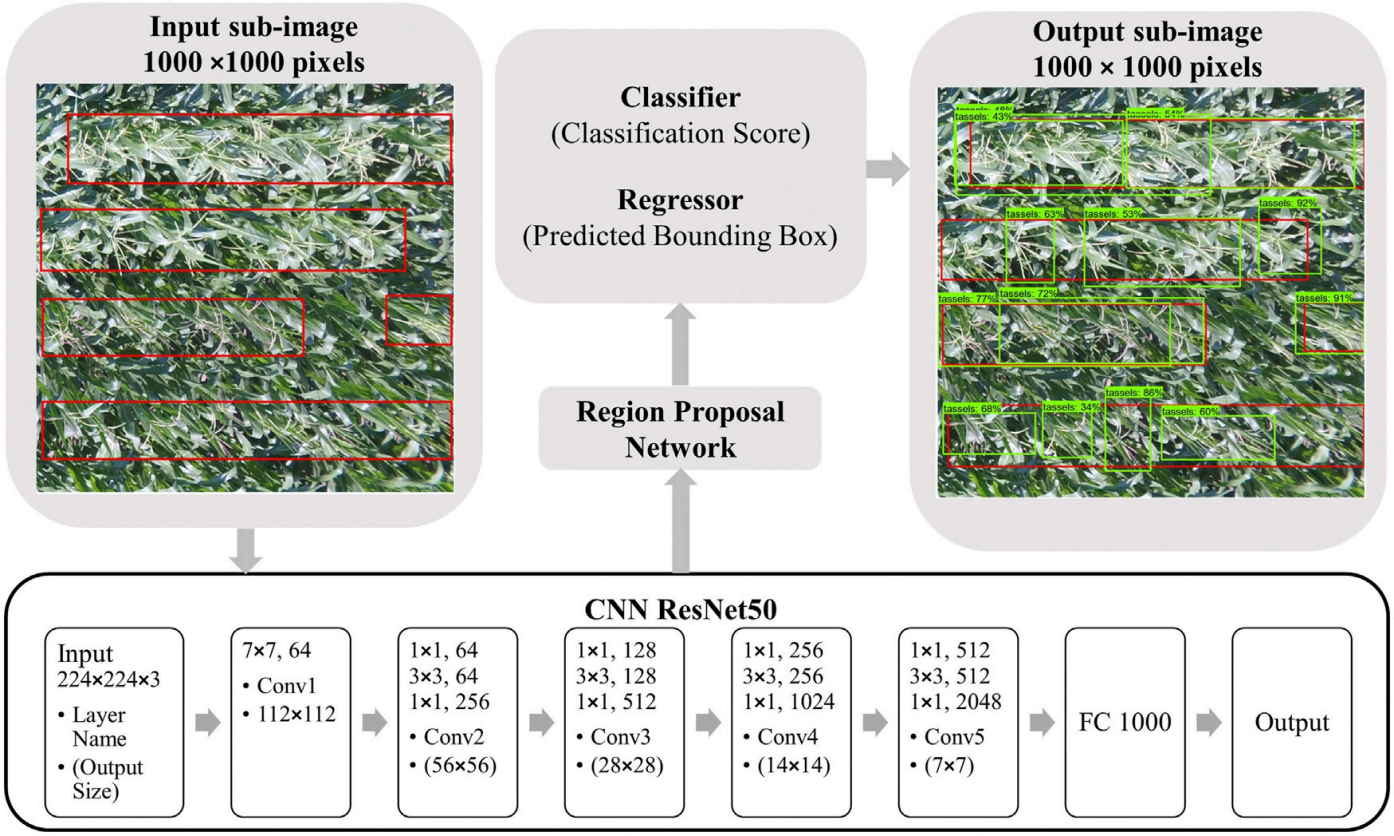
[Best viewed in color] Faster R-CNN tassel detection model trained with ResNet50 with input and output image examples.

As shown in Figure 4, the input image goes through a pretrained CNN for feature extraction to output a set of convolutional feature maps. Then a sliding window is run spatially on these feature maps to create anchors/boxes. Then, the RPN predicts the possibility of an anchor being an object, and refines the anchor. The RPN compares the anchors with the ground truth boxes and assigns a value to each anchor based on its overlap with the ground truth bounding boxes. The output of the RPN is then examined by a classifier and a regressor. The classifier will label the anchors having the higher overlaps with ground truth boxes and assigns a probability score for the predicted box containing an object, while the output of the regressor determines the coordinates of the predicted bounding box.

To train the Faster R-CNN on our dataset, we used the pretrained ResNet50 (He et al., 2016) architecture to achieve the right speed/memory/accuracy tradeoff for our tassel detection application (Huang et al., 2017). The main feature of ResNet architecture is that the use of residual path between the convolutional layers allows faster training specifically for high resolution images.

All the experimental implementations were performed using the TensorFlow Object Detection API (Huang et al., 2017), which is an open source, powerful framework used to deploy computer vision systems.

## 5 Evaluation Criteria of the Detection Models

### 5.1 Evaluation Criteria of TD-CNN

To evaluate the TD-CNN model training performance, we monitored the training cross entropy loss and validation accuracy to avoid overfitting. The trained TD-CNN model performance was evaluated based on the testing dataset (unseen data) accuracy, precision Eq. 1 and recall Eq. 2 measurements.

$$Precision=\frac{truepositive}{truepositive+falsepositive}$$ (1)

$$Recall=\frac{truepositive}{truepositive+falsenegative}$$ (2)

The precision measures the specificity, that is, how many classified tassels are actual tassels. The recall shows the sensitivity, how many actual tassels have been correctly identified.

Since the recall reflects the correctly identified tassels, we decided to pick the recall (which reflects the correctly identified tassels) as the most important metric for this study. Accordingly, some hyperparameter search was performed to obtain an optimal model. The localization performance in TD-CNN depends on the classification performance since those subimages classified as not tassel are converted to grayscale to output an image with just colored tassels. Therefore, the F1 score will directly evaluate both classification and localization in our models. The F1 score is the weighted average of precision and recall, it is calculated using Eq. 3

$$F1=2\times \frac{\text{precision}\times \text{recall}}{\text{precision}+\text{recall}}$$ (3)

### 5.2 Evaluation Criteria of Faster R-CNN

To evaluate the performance of any object detection, we need to measure the classification and localization tasks. The selected TensorFlow object detection API supports three evaluation protocols, Pascal VOC (Everingham et al., 2010), COCO (Lin et al., 2014), and Open Images (Kuznetsova et al., 2018). However, they all follow the same evaluation concept in terms of calculating the average precision (AP) value to assess the classification task. This AP was designed to evaluate the classification of several classes within the same image, which is not the case for our tassel detection task. Therefore, we created a different evaluation algorithm to measure the tassel performance based on the tassel detection specific application and based on our own observations on the output of the experiments.

For Faster R-CNN object detection case, the precision defined in Eq. 1 measures the fraction of the correct detected tassels among all the detected tassels. Whereas the recall defined in Eq. 2 reflects the fraction of relevant tassels that have been detected over the total number of the tassels present in the image. The Faster R-CNNs RPN algorithm has anchors/boxes to locate the tassels based on the ground truth according to the values of the Intersection over Union (IoU). The IoU is calculated for the bounding boxes of the ground truth and the model detection as shown in Eq. 4. Therefore, setting a threshold value of the IoU affects the true-positive, false-positive, and false-negative scores. Based on those values, the F1 score was calculated as given in Eq. 3.

$$\text{IoU}=\frac{\text{Area}\;\text{of}\;\text{Overlap}}{\text{Area}\;\text{of}\;\text{Union}}$$ (4)

**FIGURE 5:**
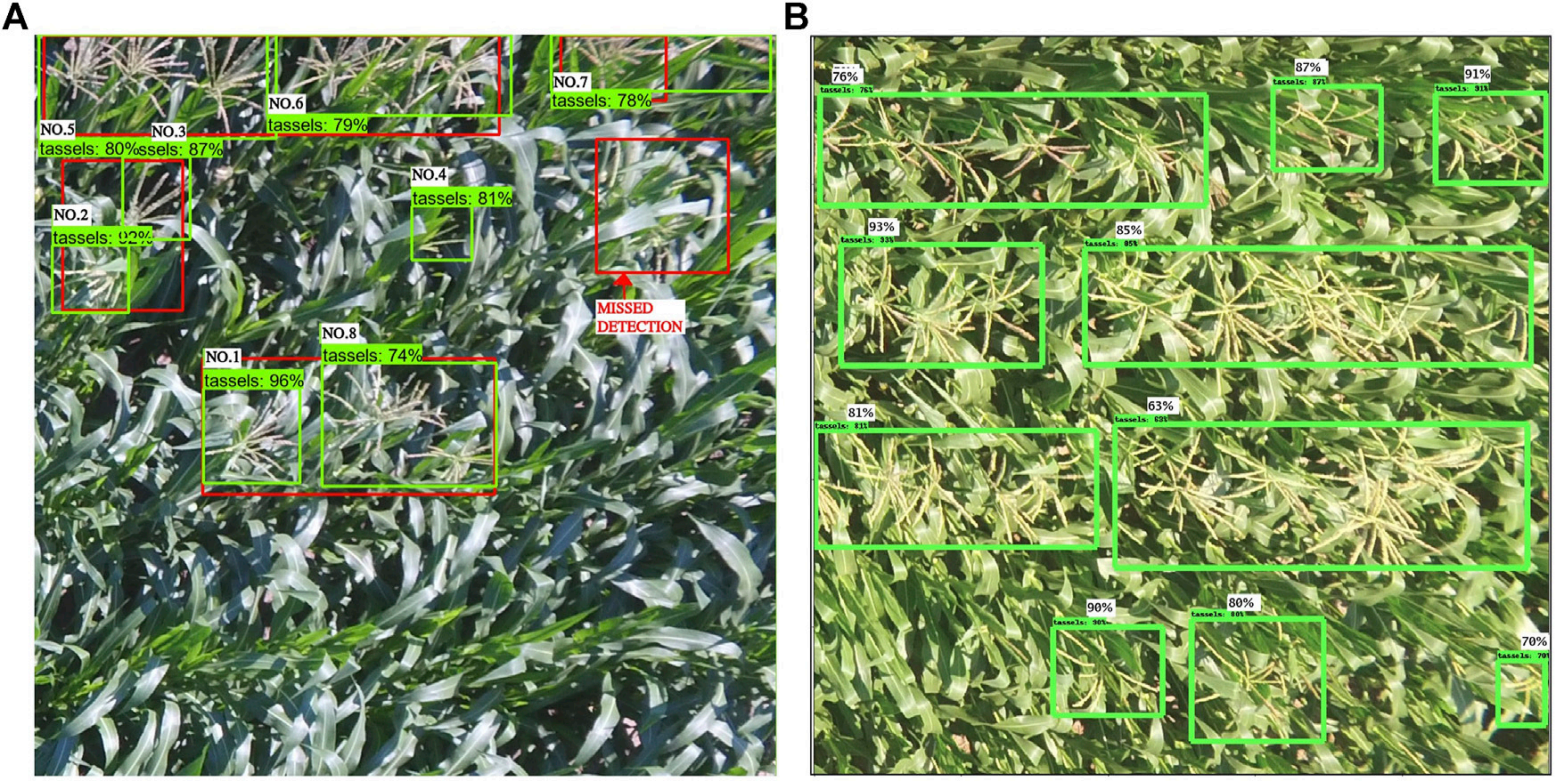
[Best viewed in color] (a) Faster R-CNN output with a low precision value (2%), although all ground truth were detected except one (green box: the model detection and red box: ground truth). (b) Faster R-CNN output all of correct tassels.

It is important to consider fitting the application while deciding how to score true positives, false positives, and false negatives since this reflects the accuracy of the model. Accordingly, some of the default thresholds of the Faster R-CNN model were altered to fit our requirement. The score threshold is the classification confidence value presented with each detected bounding box to assess the model at various level of classification confidence. Because we had one class (tassels), the trained model performed very well in classifying the tassels, and thus there was no false positive (wrong tassel detection) in the output of our model (as can be seen in the results and discussion section). Therefore, the model score threshold was set to 0.1 in our experimental setup; this low threshold allowed us to detect more tassels in the image given its accuracy score calculated by the model is greater than 0.1. Furthermore, since annotating the ground truth was done manually, some of the tassels were missed due to human error during the labeling process. As a result, the trained model detected some tassels which did not have a ground truth and therefore, counted as false positive. However, based on extensive experiments and visual evaluation analysis on all the test dataset images as can be seen in Figure 5 and Table 1, all the detection of absent ground truth bounding box were correct tassels.

**TABLE 1:** Results comparison for the detection output in Figure 5 between the default and the modified Faster R-CNN evaluation criteria.

Bounding box	Detection score (%)	Default evaluation: true positive (true), false positive (false)	Customized evaluation: true positive (true), false positive (false)
NO.1	96.36	False	True
NO.2	92.46	False	True
NO.3	87.67	False	True
NO.4	81.16	False	False
NO.5	80.74	False	True
NO.6	79.55	False	True
NO.7	78.33	False	True
NO.8	74.28	True	True

Another threshold defined in Pascal evaluation metric is the matching_iou_threshold, which is the ratio of the IoU for matching the ground truth bounding boxes to the detection boxes. This is the threshold of whether detection is to be assigned as a true positive or not. For tassel detection application, even a small partial detection of the tassels in the maize plant rows is a valuable information for the breeders. Thus, the detection box does not have to accurately match the ground truth box. This is our justification for selecting the matching_iou_threshold value of 0.3 as an optimal threshold value in our model (experimental details on evaluating different values is given in the results and discussion section). As a result, if the intersection of the model detection bounding box with the ground truth bounding box is at least 30%, this detection is considered true positive. Whereas in the Pascal evaluation metric as an example, if the performance measurement algorithm finds duplicate detection of a ground truth bounding box, they are all considered false positive.

In our tassel detection application, if the tassels in an image were next to each other, they were grouped in one box during annotation. On the other hand, individual tassels were annotated separately. This annotation methodology caused multiple size detection bounding boxes of the model on the test images. Based on this finding, we modified the algorithm to ignore the duplicate detection of the same ground truth bounding box. So, if the same ground truth has been detected more than once, the first detection will be tagged as true positive and the remaining detection are dropped.

## 6 Results and Discussion

### 6.1 Performance Evaluation of TD-CNN

**FIGURE 6:**
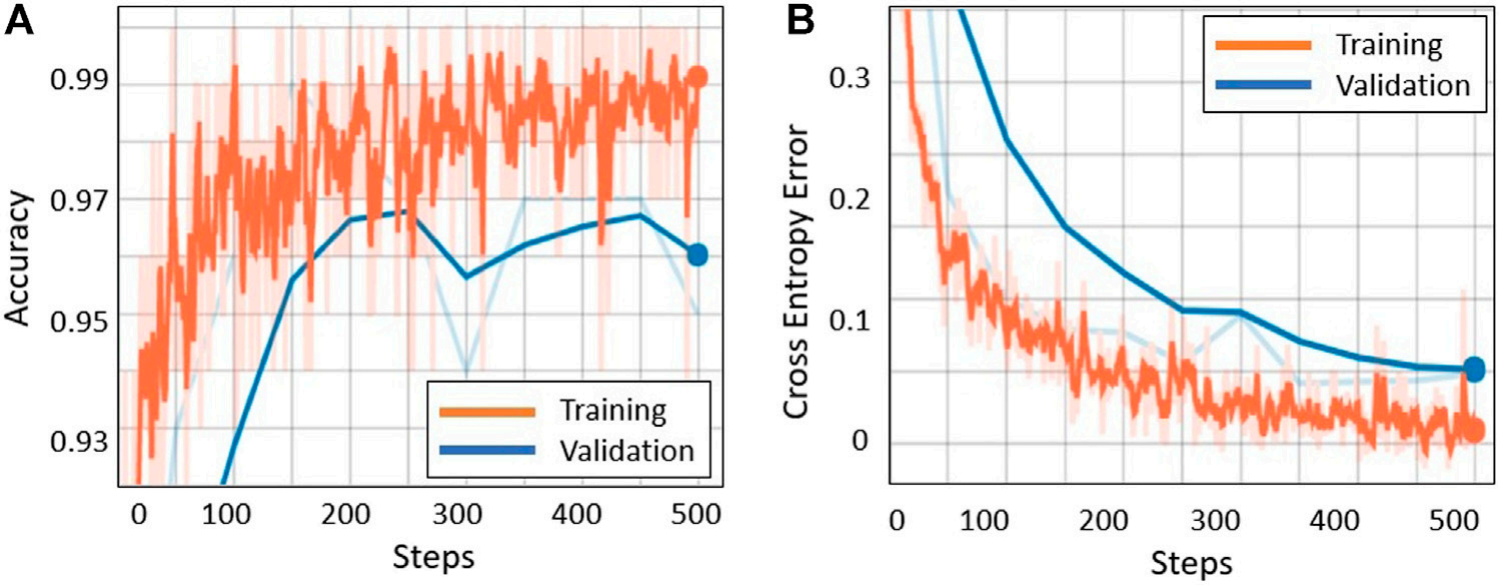
[Best viewed in color] TD-CNN (a) Training accuracy and (b) cross entropy loss vs. number of training steps.

**FIGURE 7:**
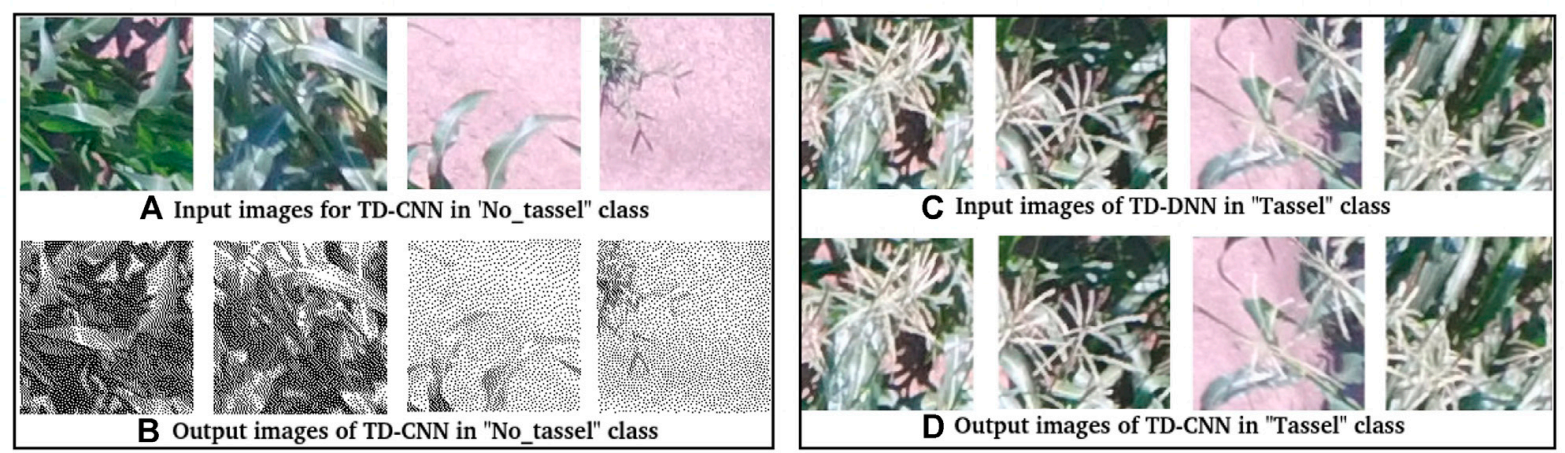
[Best viewed in color] Examples of TD-CNN model detection on the image patches. TD-CNN tassel class detection is shown in (c) and (d). The no_tassel class examples are shown in (a) and (b).

**FIGURE 8:**
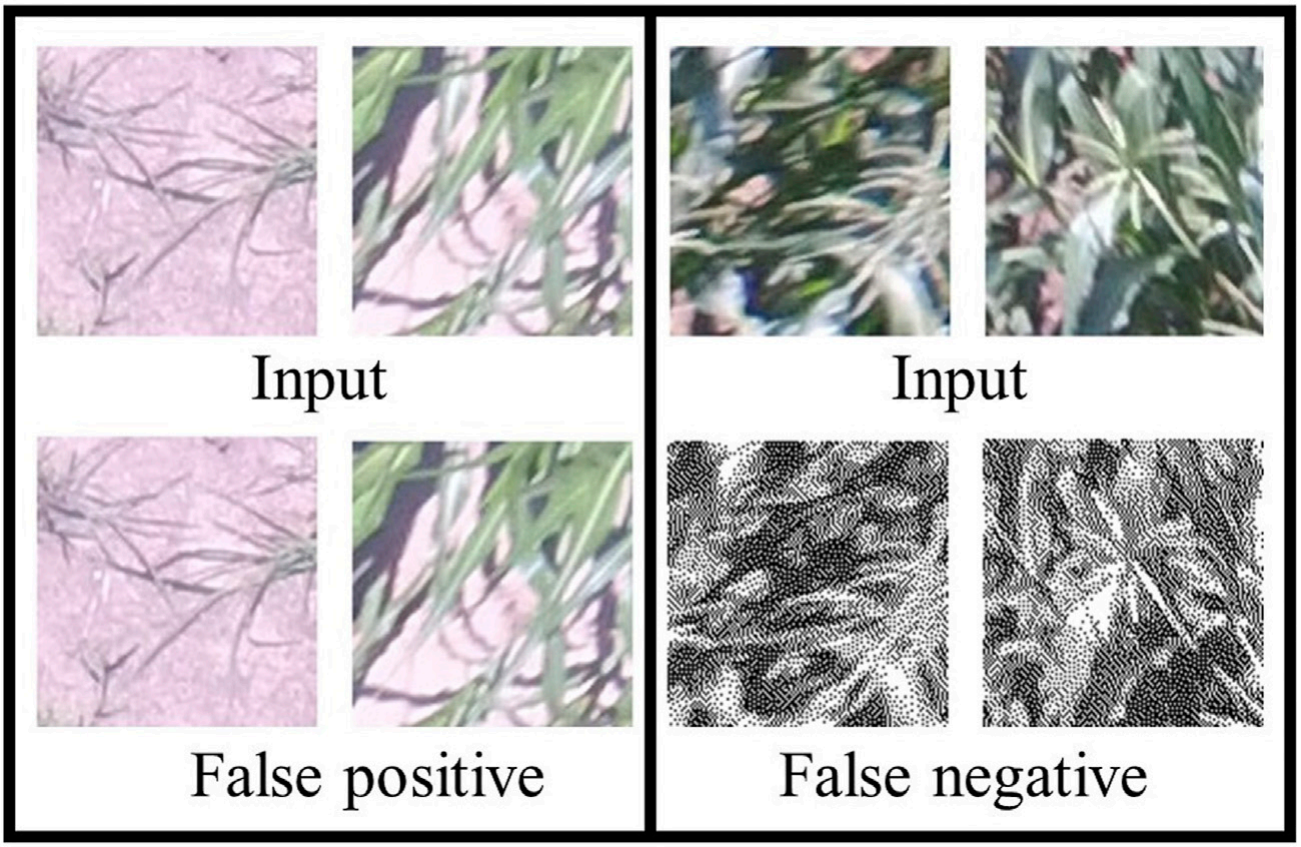
[Best viewed in color] Examples of false positives and false negatives of the TD-CNN model.

After training the model for 500 steps, we achieved 100% training accuracy with a very small cross entropy error of 0.049. Moreover, 10% of the dataset was assigned for validation purpose where an accuracy of 97% at 250 steps was achieved and then started to decrease, as shown by the blue line in Figure 6A. This is because the model is starting to overfit. However, to avoid overfitting, the best trained model used for testing was the one trained up to 250 steps since it had the highest validation accuracy. The test accuracy on the 10% test data is 96.4%, which means that the model generalizes well and is able to classify most of the “tassels” and “no_tassels” images correctly. Figure 6 show the plots of training accuracy and cross entropy error in every step. Different values for learning rates were tested, and it was observed that learning rate value of 0.01 produced the highest recall percentage of 94.6%. The use of this high learning rate might be justified by the fact that we only trained the last layer of inception v3 model, and training one layer does not require a very low learning rate. In addition to varying the learning rate, different batch size parameters were tested by fixing the best learning rate of 0.01 value obtained from the previous experiments. We varied batch sizes of 50, 100, and 200, and observed that batch size 100 produced the highest recall. Based on these tuned hyperparameters, we selected the model trained on learning rate of 0.01 and batch size of 100 to be the one used as the classification model. This chosen model achieved 97.2% precision and 94.6% recall. Based on those values, the weighted F1 from Eq. 3 was calculated to be 95.9%. Figure 7 illustrates few examples of the TD-CNN model detection on the 128 × 128 pixel patches. Some examples of false positive and false negative are shown in Figure 8.

### 6.2 Performance Evaluation of Faster R-CNN

**FIGURE 9:**
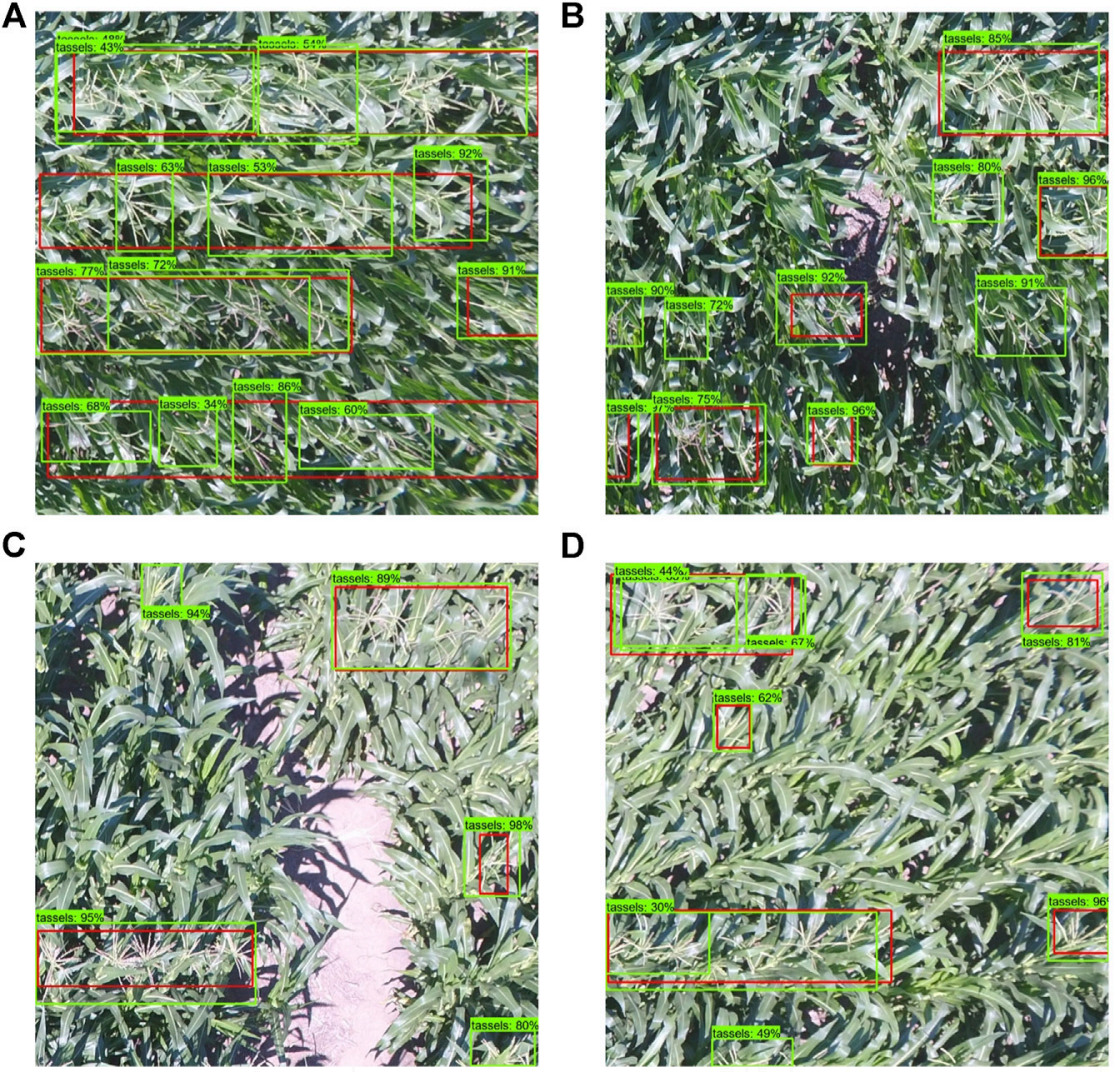
[Best viewed in color] Visualization of the trained model output on some test UAV images. The red bounding boxes refer to the ground truth, whereas the green bounding boxes refer to the model detection. Notice in (b), (c), and (d) that there are unlabeled tassels which were detected by the model.

**FIGURE 10:**
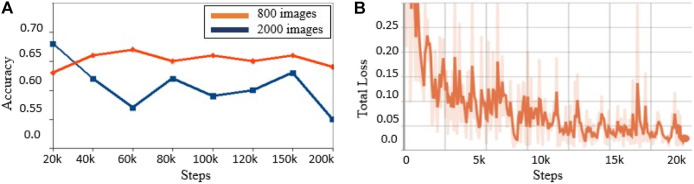
(a) Faster R-CNN model average precision values on the test data for dataset size 800 and 2000 subimages at different training steps. (b) Total losses for the Faster R-CNN best model for dataset size 2000 subimages up to 20 K training steps.

In Figure 9, we show some output images of the model performance metric on the 1000 × 1000 pixel maize field images that we used for testing (unseen during training). The red bounding box shows ground truth tassels, where the tassel class was assigned either on group of tassels or individual ones. The green bounding box shows the model predictions, where each box is allotted with a percentage score corresponding to the classification prediction confidence computed by the model. From the some statistical inspection, it can be seen that the model was able to classify and detect almost all tassels in the maize field area. The model could also detect tassels which were missed during the annotation in some cases as seen in Figures 9B–D. Moreover, the confidence level scores (associated with each classification) in our experiment are high and thus show excellent performance. We performed the Faster R-CNN training on two sizes of datasets, one with 800 images and the second with 2000 images. The training steps were varied for each set of data, and the performance of the model was evaluated on the test images. Figure 10 summarizes the average precision on the unseen testing set for the trained model with each dataset size. The average precision values in Figure 10A are based on the default performance measurement metrics before adjusting the threshold as discussed later in this section. The optimal model with the highest average precision of 0.68 was selected as the inference model for the detection system. Figure 10B demonstrates the total losses graph generated for Faster R-CNN ResNet50 by TensorBoard while training the best model in this task.

However, we noticed that the calculated average precision is not reflecting the true performance of the model output. Examining the images which had the worst precision score of just 2.5% (Figure 5), most tassels were successfully detected except of one false positive and one true negative. Table 1 shows the cumulative assigned average precision for each bounding box detected by the default Pascal evaluation metric script. Each bounding box was associated with a detection score representing the likelihood of the box containing an object. This image has eight detected bounding boxes of them correctly detecting the tassels, but only one detected bounding box was counted as true positive by the default evaluation criteria, which resulted on the average of one eighth precision for the entire output. The reported recall value for the image is 0.2, due to the missed one of the total five ground truth–bounding boxes. Since the nature of tassel distribution in the maize field is variant, this caused an inconsistent labeling procedure where some tassels were grouped together in one annotated box and some were individually annotated. This observation proved the fact that the default setting of counting the duplicated detection as false positive falsely affected the correct tassel detection system performance measurement.

From the above discussion, it follows that the default Pascal evaluation metric scripts do not show correct performance for our tassel detection application. Therefore, we modified the default performance measurement metric scripts to suit our tassel detection application. The default Pascal evaluation metric scripts were modified to ignore any duplicate detection of the same ground truth–bounding box instead of being a false-positive one. In addition, the iou_matching and the classification min_score thresholds were varied to help concretely measure the performance of the application for detecting the tassels. The iou_matching threshold decides the minimum accepted area of a detection compared to the ground truth area. All values below 0.3 did not affect the average precision, which means that all the detection are covering at least 30% of the ground truth–bounding box area. Another default threshold which affect the evaluation performance is called the classification minimum score threshold. It reflects the probability of the classified object, tassel in our case. As shown in Figure 5, all the classified objects were correctly classified as tassels since we only had one class. So, we lowered this threshold to 0.1 to allow the model to detect as much classified tassels as possible. Finally, setting the iou_matching threshold to 0.3 and the minimum classification score to 0.1 improved the Pascal object detection average precision to 83.82%. To assess this decision, the standard classification precision and recall metrics were computed as given in Eqs 1, 2. The inference model has an average classification precision of 97.64%, and an average recall of 98.32%. The F1 score was calculated using Eq. 3 with the value of 97.98%.

### 6.3 Discussion

In this study, we were able to detect tassels from high-resolution images with high detection accuracy using two different deep learning models. We implemented our own framework for object detection using the existing “regular patch–based classification” method. And we modified the evaluation criteria of the off-the-shelf Faster R-CNN model to fairly measure the performance of tassel detection. We demonstrated how deep learning models can be used to automate many breeding processes in agriculture, and tassel detection is just one example. We consider one of the major challenges and contribution of our work is related with the relatively low quality of the original images. Most of the other similar work on the CNN-based maize tassel detection was with the original images in much higher quality, that is, higher spatial and radiometric resolutions so that the tassels are more clearly seen and more easily distinct with the maize canopy and soil background. However, in real applications, data collected may not always be in high and desired quality. Hence, we think it is worth to investigate and benchmark the potential of how the relatively lower quality images can do on the tassel detection. Another major contribution of this work is beyond the pure model performance in terms of accuracy and implicates on future work on other datasets or applications. Table 2 shows the relative differences in accuracies and computational resources requirements or time consumptions between the two models. With a little bit of sacrificing the detection accuracy, the customized patch-based TD-CNN gains a lot more on the training time.

**TABLE 2:** Comparison of the TD-CNN model with patches of size 128×128, and the off-the-shelf Faster R-CNN object detection method with subimage size of 1000×1000 on the tassels dataset.

Method	Complexity	Model training speed (GPU)	F1 score (%)	Output visualization
TD-CNN	Composed of one classification Inception v3 neural network	1hour	95.9	Grayscaling
Faster R-CNN	Composed of three neural networks: Feature ResNet50 network, region proposal network (RPN), and detection network	≈16hours	97.9	Bounding box

**FIGURE 11:**
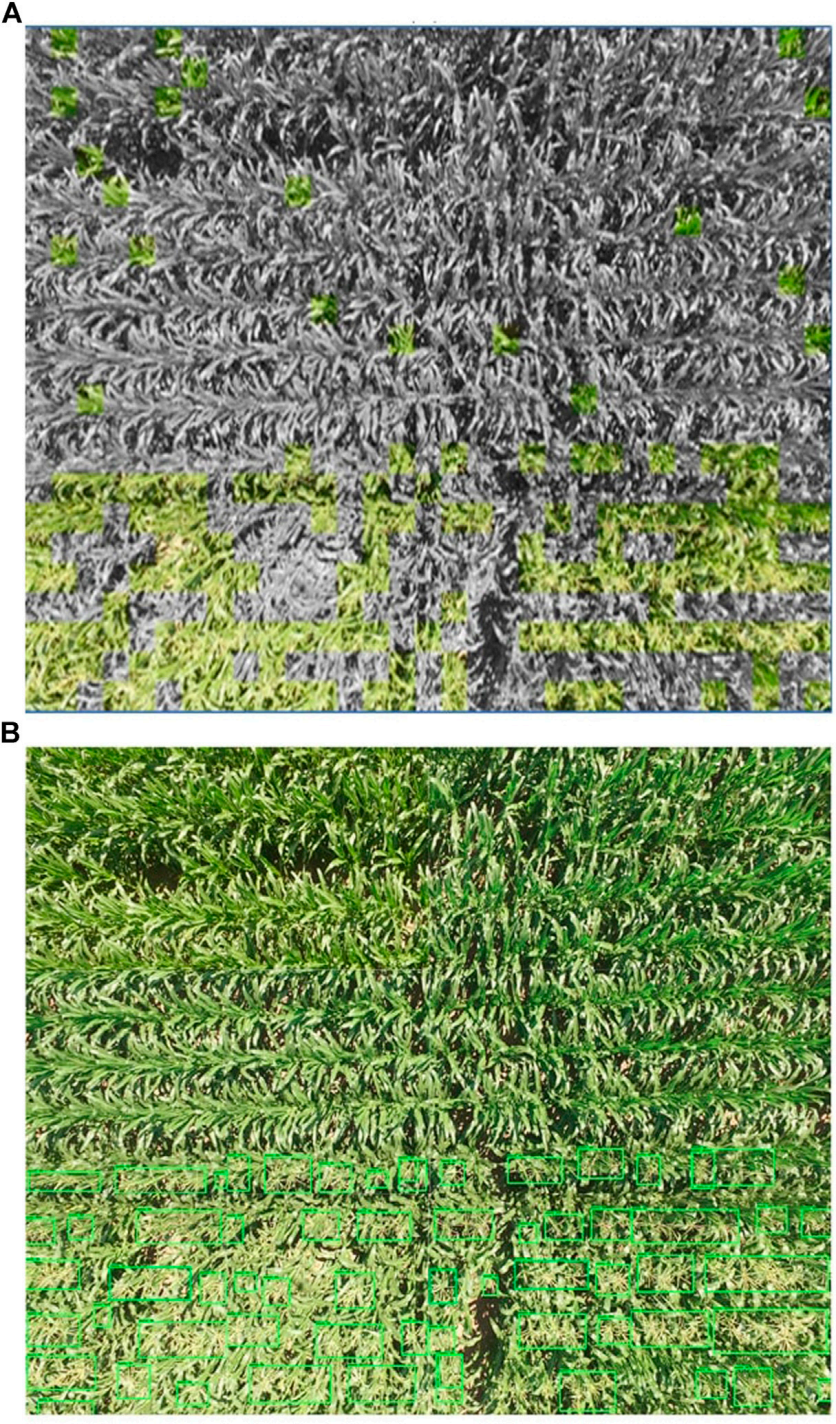
Visualizing an example of tassel detection outputs of the same full UAV image of size 3000×4000 pixels for both (a) TD-CNN, the grayed areas are no_tassels, the colored areas are “tassels.” (b) Faster R-CNN, the green boxes are tassels.

Table 2 shows the comparison of our proposed TD-CNN approach with the off-the-shelf Faster R-CNN for tassel detection in UAV images. The TD-CNN framework is relatively easier to implement and faster to train, but it had lower recall rate which caused few false localization of the no_tassel regions. In term of the model architecture complexity, the Faster R-CNN requires advanced knowledge in deep learning for the user to understand its architecture, but it produces an accurate inference model that can correctly identify tassels from UAV images. The TD-CNN uses the grayscaling technique to locate tassels within the image, while the Faster R-CNN draws a bounding box around the detected tassels. Moreover, the F1 score of Faster R-CNN is higher than that of the TD-CNN model, which makes the Faster R-CNN a better option that generalizes well on new data and detects almost all the tassels correctly. Figure 11 shows one example of the tassel detection on one of the UAV images with both TD-CNN and Faster R-CNN. Figure 11A shows some false positives (the red boxes) in the TD-CNN output, where the Faster R-CNN has proven to have no false positives at all.

As future work, we consider improving the performance of the TD-CNN model by training with more data, and trying different CNN architecture models for the transfer learning stage. Another future work is to deploy the detection models on a drone for real-time tassel detection.

## 7 Conclusion

The use of deep learning algorithm for tassel detection from low-altitude UAV imagery is investigated in this study. We introduced a novel tassel detection framework based on deep learning, called the tassel detection CNN. Moreover, we demonstrated the feasibility of using Faster R-CNN for tassel detection in UAV images. Both models were able to detect tassels with high accuracy despite the challenges of relatively lower spatial resolution and clarity due to motion blur compared with imagery collected from proximal sensing from ground-based platforms. Several evaluation criteria have been modified to get the best performance for tassel detection. We modified the default Pascal evaluation algorithm which assigns a false positive tag to the duplicate detection. The duplicate detection was rather ignored to reduce the negative unnecessary impact on the performance of our tassel detection model. The F1 score for both methods is high, but the recall is much higher for the Faster R-CNN model since it had not produced any false positives at all.

## Data Availability

The raw data supporting the conclusion of this article will be made available by the authors, without undue reservation.
